# PVL overexpression due to genomic rearrangements and mutations in the *S. aureus* reference strain ATCC25923

**DOI:** 10.1186/s13104-017-2891-3

**Published:** 2017-11-07

**Authors:** Bettina Stieber, Artur Sabat, Stefan Monecke, Peter Slickers, Viktoria Akkerboom, Elke Müller, Alexander W. Friedrich, Ralf Ehricht

**Affiliations:** 1 Alere Technologies GmbH (Abbott Rapid Diagnostics), Jena, Germany; 2 InfectoGnostics Research Campus, Jena, Germany; 3 Department of Medical Microbiology, University of Groningen, University Medical Center Groningen, Groningen, The Netherlands; 4 Institute for Medical Microbiology and Hygiene, Technische Universität Dresden, Dresden, Germany

**Keywords:** *Staphylococcus aureus*, Panton-Valentine leukocidin (PVL), Protein expression, Sequence mutation

## Abstract

**Objective:**

ATCC25923 is a *Staphylococcus aureus* strain that is positive for the Panton Valentin leukocidin. It has been used for decades as reference strain. We observed that two separately maintained clones of ATCC25923 (“G477 and G478”) differed grossly in the expression of this toxin. For that reason, both clones were sequenced using an Illumina MiSeq instrument. After assembling, the final sequences were analyzed and mapped to a previously published ATCC25923 sequence (GenBank CP009361) using bl2seq from the NCBI Blast2 package.

**Results:**

The genomes of G477 and G478 size 2778,859 and 2792,213 nucleotides, respectively. Both genomes include a circular plasmid of 27,490 nucleotides. The sequence of the G477 chromosome maps nearly exactly to CP009361. G478 has a slightly larger size because of the presence of an additional transposable element tnp13k. The second copy of that tnp13k element is located in an intergenic region between the genes *mazF* and *rsbU*. The sequences of the ATCC25923 clones G477 and G478 differ mainly in the insertion of a second tnp13k element between the genes *mazF* and *rsbU*. That insertion may lead to a different transcription of that genome region resulting in upregulation of the expression of the Panton-Valentine leukocidin in the ATCC25923 clone G478.

**Electronic supplementary material:**

The online version of this article (10.1186/s13104-017-2891-3) contains supplementary material, which is available to authorized users.

## Introduction

*Staphylococcus aureus* (*S. aureus*) is a facultative pathogen that can colonize or infect humans as well as animals. The virulence of this bacterium is influenced by various virulence factors which are frequently localized on mobile genetic elements allowing horizontal gene transfer between strains. One example is the Panton-Valentine leukocidin (PVL). PVL is an exotoxin encoded by the genes *lukF*-*PV* and *lukS*-*PV*. The two genes are co-located on a prophage, and co-expressed [1] to form polymeric pores in leukocyte membranes and to trigger apoptosis. The toxin is associated with skin and soft tissue infections as well as with life-threatening diseases such as necrotizing pneumonia and necrotizing fasciitis [2].

ATCC25923 is a methicillin susceptible and PVL-positive laboratory strain. It belongs to clonal complex (CC) 30 and sequence type (ST) 243 that differs from parental ST30 by a single nucleotide mutation in the *glpf* gene. ATCC25923 was isolated in Seattle in 1945, i.e., before penicillin or other antibiotics widely entered clinical practice. Due to the absence of any resistance genes from this strain, it is frequently used as a susceptible control during antibiotic susceptibility tests. For that purpose, many laboratories have cultured and maintained this strain for decades and this might have given rise to locally distinct clonal lineages over time. One such lineage, purchased through Remel Inc., Lenexa, KS under license of the American Type Culture Collection, has already been sequenced in 2014 [3]. This sequence was placed in GenBank (Accession Numbers CP009361 for its chromosome and CP009362 for a plasmid) [http://www.ncbi.nlm.nih.gov/nuccore/CP009361; http://www.ncbi.nlm.nih.gov/nuccore/CP009362].

In our laboratory, two other local clones of ATCC25923 were investigated that differ remarkably in the expression and secretion of PVL as demonstrated in a previous study [4]. In order to identify genomic changes and/or mutations leading to this noticeable difference, the genomes of both ATCC25923 clonal lineages were sequenced and analyzed.

## Main text

### Materials and methods

#### Strains

One clone of ATCC25923 was maintained, used and passaged for at least 12 years at the Institute for Medical Microbiology and Hygiene, TU Dresden, Germany. This clone, named G478 hitherto, was found to be a PVL hyper-producer. A second clone, G477, was obtained from another bacteriological laboratory, Bioscientia MVZ Saarbrücken (courtesy of Dr. Stefan Weber).

#### Protein measurements

PVL production was measured using tube-mounted antibody arrays (Alere Technologies GmbH; cat. nr. 201010719). The method was previously described in detail [4]. In short, strains were cultured for 18 h in liquid broth as described by Kato and Noda [5]. Supernatants were added to arrays, on which dilution series of LukF-PV specific monoclonal antibodies were spotted and covalently immobilized. Subsequently, a biotin-labelled secondary antibody, streptavidin-horseradish-peroxidase and a precipitating dye (reagents from Protein Binding Kit by Alere Technologies; cat. nr. 245500100) were added. PVL concentrations were determined by mapping the resulting spot intensities to calibration curves that resulted from experiments with known concentrations of recombinant and purified LukF-PV. Experiments were repeated five times (G477) or six times (G478) using different batches of growth media.

#### DNA-array-based typing

Clones were genotyped by DNA-Microarrays (*S. aureus* Genotyping Kit 2.0 kit, Alere Technologies; cat. nr. 245200096). Microarray procedures have been previously described in detail [6, 7]. In addition, probes for plasmid-borne heavy metal resistance genes were used (see Additional file 1).

Furthermore, the sequences of the probes on the microarray were mapped against the previously published ATCC25923 sequence (CP009361 + CP009362) facilitating the prediction of its hybridization pattern.

#### Sequencing and analysis

One colony forming unit from visibly pure culture of each isolate was selected for whole genome sequencing. Genomic DNA was extracted by enzymatic lysis using the enzymes and buffers provided with the *S. aureus* Genotyping Kit 2.0 kit (Alere Technologies; cat. nr. 245200096) and the DNeasy Blood and Tissue Kit (Qiagen; cat. nr. 69506) according to the manufacturers’ instructions. A library was prepared using the Nextera XT kit (Illumina; cat. nr. FC-131-1024) according to the manufacturer’s instructions. Briefly, the purified genomic DNA quantified with a Qubit dsDNA BR (broad range; Thermofisher; cat. nr. Q32850) assay kit and a Qubit 2.0 Fluorimeter (Thermofisher) was diluted to 0.2 ng/µl and then the DNA concentration was measured again using this time Qubit dsDNA High Sensitivity assay kit (Thermofisher; cat. nr. Q32851). A total of 1 ng of DNA was tagmented at 55 °C for 5 min. PCR amplification to introduce Illumina index sequences was performed in PCR strip tubes in a BioRad T100 thermocycler. The size distribution of fragments was estimated with a 2200 TapeStation using the Agilent D1000 High Sensitivity kit (Agilent; cat. nr. 5067-5585) according to the manufacturer’s instructions. Fragments of 200 to 1000 bp were obtained. The library DNA fragments were size selected and purified using Agentcourt AMPure XP beads (Beckman Coultier Genomics; cat. nr. A63881). The indexed libraries were normalized, pooled and loaded onto an Illumina MiSeq reagent cartridge using MiSeq reagent kit v3 (Illumina; cat. nr. MS-102-3003) and 600 cycles. The paired-end 2× 300 bp sequencing was run on an Illumina MiSeq sequencer. The reads were assembled de novo utilizing the SeqMan NGen 12.1.0 software with the following assembly parameters: Match Size: 31; Match Spacing: 50; Minimum Match Percentage: 93; Match Score: 10; Mismatch Penalty: 20; Gap Penalty: 30; Max Gap: 6; Minimum read length: 150; Minimum sequences per contig: 100. The resulting contigs were ordered by using the Mauve Contig Mover [8] relative to the ATCC25923 reference genome (GenBank Accession Number CP009361). End-to-end alignment of contigs to find overlap between adjoining contigs was accomplished using the SeqMan Pro 12.1.0 software (DNAStar). It allowed merging the majority of the contigs in the draft genomes. Subsequently the gaps were closed by an extension of the contig ends using the reference-guided genome closure protocol and the SeqMan NGen software followed by merging contigs in the SeqMan Pro software.

Remaining gaps between contigs were closed by PCR amplification with primers flanking the gaps followed by Sanger or MiSeq sequencing (see Additional file 2).

The assembly files with the complete and closed genomes were exported as fasta files and used in further analyses.

#### Software procedures and analysis

The final sequences of G477 and G478 were mapped to the published ATCC25923 sequence (CP009361; [http://www.ncbi.nlm.nih.gov/nuccore/CP009361]) using bl2seq from the NCBI Blast2 package [9]. In order to identify single nucleotide polymorphisms (SNPs), co-linear regions were compared position by position with an in-house python script.

## Results and discussion

### Protein measurements

During a previous study [4] it was shown that G478 produced more PVL than any other strain tested with a mean of 41,000 ng/ml and a median of 22,000 ng/ml (range from 10,000 to 151,000 ng/ml). G477 yielded a mean and median of 1300 ng/ml (range from 400 to 2400 ng/ml), which was more in accordance to other CC30 strains tested during that study [4].

### DNA-array-based typing

G477 and G478 appeared as typical CC30-MSSA as previously described [6] and were identical in all features covered by the microarray. They presented with PVL genes and the *egc* enterotoxin gene cluster. They lacked all other enterotoxin genes, *tst1* and genes associated with beta-haemolysin-converting phages (*sak, scn, chp, sea, see*). Genes associated with antibiotic resistance were absent, but some genes for heavy metal resistances (*mco, copA2, arsB, arsC* and *cadD*) were detected using a new set of probes.

The predicted hybridization pattern for CP009361 + CP009362 fully matched the results observed for G477 and G478 (see Additional file 1).

### Sequencing

Sequencing yielded an average genome coverage of 146× (minimum 7× and maximum 2188×) and 355× (minimum 11× and maximum 4648×) for G477 and G478, respectively. The complete genome of G477 was assembled from the sequencing reads, yielding a circular bacterial chromosome (size 2,778,859 nt) and a circular plasmid (size 27,490 nt). The assembled genome of G478 consists of a circular bacterial chromosome (size 2,792,213 nt) and a circular plasmid (size 27,490 nt) (see Table 1). The circular chromosomal and plasmidic sequences were linearized at the same positions as GenBank entries CP009361.1 and CP009362.1. The plasmid sequences of G477 and G478 were proven to be 100% identical over their entire length, whereas the chromosomal sequences differ by about 13,500 nt in size. The shorter chromosome of G477 is nearly equal in size to CP009361.1 (difference only 5 nt).

**Table 1:** Full genome sizes of ATCC25923 derivatives

Isolate	Molecule	Length (nt)
G477	Chromosome	2,778,859
G477	Plasmid	27,490
G478	Chromosome	2,792,213
G478	Plasmid	27,490
CP009361.1	Chromosome	2,778,854
CP009362.1	Plasmid	27,491

**Fig. 1:**
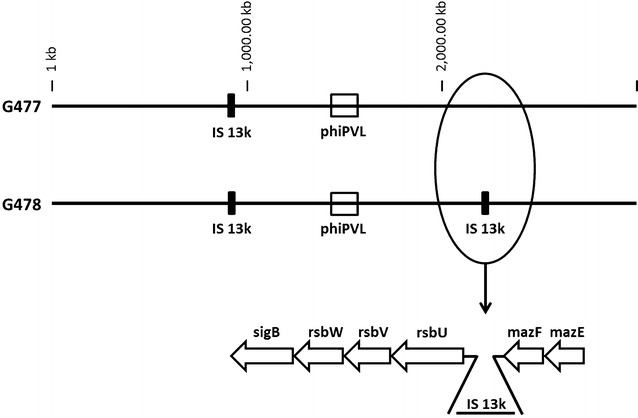
Insertion of the additional tnp13k element in G478. The insertion site of the additional tnp13k element in the *S. aureus* clone G478 of the strain ATCC25923 near position 2,108,925 nt. The insertion is located in an intergenic region between the genes *mazF* and *rsbU*

The increased size of G478 is caused by a single insertion event where an additional stretch of about 13,500 nt is integrated near position 2,108,925 (see Fig. 1). This corresponded to a gap between G478 contigs that was closed by sequencing of a PCR product. This additional stretch of DNA was identified to be an exact copy of a transposable element tnp13k which is present with a single copy in the G477 and CP009361.1 chromosomes near position 989,942.

Comparison of the G477 and G478 chromosomal sequences allows mapping the insertion site precisely. The insertion site has a very low GC content and the insertion causes a duplication of 3 nucleotides (TAA). A similar observation has been made by Schijffelen et al. [10] who found two copies of the 13 K conjugative transposon in the strain S0385 (CC398-MRSA-V), GenBank AM990992. However, the sequences of the two copies differed substantially from each other in that case [10].

Beside the duplication of tnp13k, the chromosomes of G477 and G478 are co-linear and differ by only 9 SNP. No further insertions or deletions were found.

These 9 SNPs are listed in Table 2. SNPs 1 and 6 are located in intergenic regions, and thus they cannot be associated with any changes in amino acid sequences. SNPs 2 and 5 do not cause a change in amino acid sequences of the respective gene products and thus they cannot influence metabolic pathways such as, e.g., toxin expression. SNPs 3, 4, 7, 8 and 9 cause changes in amino acid sequences, but the functions of the proteins in question (fatty-acid-binding protein, homoserine kinase, RNA binding proteins and isopentenyl-diphosphate delta-isomerase; see Table 2) do not suggest that these changes might be related to the different PVL expressions in the two clones.

**Table 2:** Single nucleotide polymorphisms (SNPs) of G478

SNP	G477 position	G478 position	Locus	Gene	Function	NA change (G477 to G478)	AA change (G477 to G478)
1	464,504	464,504	Intergenic	Between recR and rrs, polyT	(Sequencing error?)	G to T	None
2	705,579	705,579	CDS	yflS	Putative malate transporter	T to A	None (silent mutation)
3	768,873	768,873	CDS	degV1	Putative fatty-acid-binding protein	G to C	AA 179: A to P
4	1,318,286	1,318,286	CDS	thrB	Homoserine kinase	T to C	AA 153: M to T
5	1,333,827	1,333,827	CDS	sbcC	Nuclease Sbc subunit C	C to T	None (silent mutation)
6	2,051,482	2,051,482	Intergenic	Between AIO21657 and AIO21658	Mobile element	G to A	None
7	2,174,003	2,187,357	CDS	Q5HE59	Putative RNA binding protein	G to A	AA 454: T to V
8	2,174,004	2,187,358	CDS	Q5HE59	Putative RNA binding protein	T to C	AA 454: T to V (see above)
9	2,373,530	2,386,884	CDS	fni	Isopentenyl-diphosphate delta-isomerase	G to A	AA 40: S to L

The sequences of integrated PVL-phage and PVL genes were also compared for CP009361, G477 and G478. Data showed identical sequences in all three ATCC25923 derivatives. Therefore, neither the PVL genes (*lukF*-*PV* and *lukS*-*PV*) nor the phage-located promotor could be the reason for the different PVL expressions in G477 and G478 indicating that differences in the core genome might be responsible. This also ruled out the possibility that the apparent difference in PVL expression was caused by different binding affinities of the antibodies due to slightly different sequences of LukF-PV.

Thus, special attention was paid to the additional tnp13k element in G478 which is inserted into the intergenic region between the core chromosomal genes *rsbU* and *mazF* (Fig. 1).

*MazF* is part of the chromosomally encoded and well characterized MazE (antitoxin)/MazF (toxin) system. It encodes an endoribonuclease which specifically cleaves mRNA at the pentad sequence UACAU. This sequence was identified in several genes for virulence factors of *S. aureus*, e.g., in the gene of SraP, which mediates the adhesion of the bacteria to host cells [11]. A further study showed that MazF cuts also *spa* and *rsbW* at UACAU sites whereas the expression of these proteins was unaffected by the mRNA cleavage. These findings suggest that MazF plays a role in *S. aureus* gene regulation [12].

The other neighboring gene, *rsbU,* encodes a phosphatase that positively controls sigmaB, which itself downregulates the expression of *agr*. Agr is a well-known regulator of *S. aureus* virulence and a transcriptional factor that plays a central role in stress response and persistence of infection. Moreover, RsbU is able to dephosphorylate a global transcriptional regulator, MgrA, controlling expression of virulence genes [13–15].

The insertion site of the additional 13K element into the intergenic region between MazF and rsbU may affect the transcription of the neighboring genes (see Fig. 1). A previous study showed the transcription of a large mRNA starting at the promotor of the *mazE* gene and then continuing through *mazF, rsbU, rsbV, rsbW* and *sigB* (a 3.7 kb transcript). *MazE, mazF* and *rsbU* are transcribed together, despite that there is a 350-nt intergenic region between them [16].

However, there are two further promotors immediately upstream of *rsbU* and *rsbV*, respectively, as well as a weak transcriptional terminator downstream of *mazF*. Presumably, two further transcription products of different length are formed [16]. The tnp13k element was inserted 195 nt upstream from the *rsbU* translation start site and might interfere with transcriptional activation of the *rsbU* promoter leading to up-regulation of PVL expression.

The analyzed sequences of the ATCC25923 clones G477 and G478 differ mainly in the additional insertion of a second copy of the tnp13k element in G478. The insertion between the genes *rsbU* and *mazF* seems to affect the usual transcription and leads to the previously detected up-regulation of the PVL expression and secretion in G478.

## Limitations

It was assumed that the insertion of a tnp13k element upstream from the *rsbU* translation start site lead to an up-regulation of PVL expression. However, the exact pathway still needs to be determined. Further evidence for this hypothesis should be added by deleting the second tnp13k element from the G478 genome followed by a measurement of PVL expression, or by introducing such an insert at the same localization into another well-characterized strain.

## Additional files

**Additional file 1.** (1) Core genomic markers, sorted by position in genome; (2) Mobile genetic elements; phage-associated markers; (3) Mobile genetic elements; other virulence markers; (4) Mobile genetic elements; SCC-associated markers; (5) Mobile genetic elements; other resistance markers.

**Additional file 2.** Primers used for filling the gaps between the NGS contigs by sequencing of PCR products.

## Abbreviations

A: adenine (in DNA sequences), alanine (as amino acid)
AA: amino acid
ATCC: American Type Culture Collections
Bp: base pair(s)
C: cytosine
cat. nr.: catalogue number
CC: clonal complex
DNA: deoxyribonucleic acid
G: guanine
Kb: kilobase, 1000 nucleotides
L: leucine
M: methionine
mRNA: messenger ribonucleic acid
MRSA: methicillin-resistant *Staphylococcus aureus*
MVZ: “Medizinisches Versorgungszentrum”, outpatient health centre (in Germany)
NA: nucleic acid
NCBI: National Center for Biotechnology Information
ng/ml: nanogram per milliliter
NGS: next-generation sequencing
Nt: nucleotides
P: proline
PCR: polymerase chain reaction
PVL: Panton-Valentine leukocidin
S: serine
*S. aureus*: *Staphylococcus aureus*
SCC: Staphylococcal cassette chromosome
SNP: single nucleotide polymorphism
ST: sequence type
T: thymine (in DNA sequences), threonine (as amino acid)
V: valine
