# Supplementary material for: PVL overexpression due to genomic rearrangements and mutations in the S. aureus reference strain ATCC25923
Source: BMC Res Notes. 2017 Nov 7;10:576. doi: 10.1186/s13104-017-2891-3 (PMC5678758; doi:10.1186/s13104-017-2891-3)
Supplement: Supplementary file 1 — Additional file 1. (1) Core genomic markers, sorted by position in genome; (2) Mobile genetic elements; phage-associated markers; (3) Mobile genetic elements; other virulence markers; (4) Mobile genetic elements; SCC-associated markers; (5) Mobile genetic elements; other resistance markers. [file 13104_2017_2891_MOESM1_ESM.docx]

**Additional file 1. Array hybridisation profiles for ATCC25923 clones**

NEG = negative, AMB = ambiguous, **POS** = positive.

1. **Core genomic markers, sorted by position in genome**

| **GENE** | **EXPLANATION** | **ALLELE** | **Predicted profile for ATCC 25923 (GenBank CP009361+**  **362)** | **Array hybridization data for ATCC25923-G478** | **Array hybridization data for ATCC25923-G477** |
| --- | --- | --- | --- | --- | --- |
| ***ent*CM14** | enterotoxin-like protein ORF CM14 | - | NEG | NEG | NEG |
| ***seh*** | enterotoxin H | - | NEG | NEG | NEG |
| ***spa*** | staphylococcal protein A | - | **POS** | **POS** | **POS** |
| ***capH*** | capsular polysaccharide synthesis enzyme | capH5 | NEG | NEG | NEG |
|  |  | capH8 | **POS** | **POS** | **POS** |
| ***capI*** | capsular polysaccharide biosynthesis protein | capI8 | **POS** | **POS** | **POS** |
| ***capJ*** | O-antigen polymerase | capJ5 | NEG | NEG | NEG |
|  |  | capJ8 | **POS** | **POS** | **POS** |
| ***capK*** | capsular polysaccharide biosynthesis protein | capK5 | NEG | NEG | NEG |
|  |  | capK8 | **POS** | **POS** | **POS** |
| ***lmrP*** | hypothetical protein, similar to integral membrane protein LmrP | lmrP (OtherThanRF122) | **POS** | **POS** | **POS** |
|  |  | lmrP (RF122) | NEG | NEG | NEG |
|  |  | lmrP (OtherThanRF122) | **POS** | **POS** | **POS** |
|  |  | lmrP (RF122) | NEG | NEG | NEG |
| **Q9RL82** | putative protein  island between hsdR and oppF2 | Q9RL82 (consensus) | NEG | NEG | NEG |
|  |  | Q9RL82-CC10/361 | NEG | NEG | NEG |
|  |  | Q9RL82-CC8 | NEG | NEG | NEG |
| ***CoA*** | coagulase | - | **POS** | **POS** | **POS** |
| ***esxA*** | secreted virulence factor | - | **POS** | **POS** | **POS** |
| ***esxB*** | secreted virulence factor | - | NEG | NEG | NEG |
| ***setC*** | staphyl. exotoxin-like protein | setC / selX | NEG | NEG | NEG |
| ***ssl01*** | staphylococcal superantigen-like protein 1 | ssl01/set6 (COL) | NEG | NEG | NEG |
|  |  | ssl01/set6 (MRSA252) | **POS** | **POS** | **POS** |
|  |  | ssl01/set6 (Mu50+N315) | NEG | NEG | NEG |
|  |  | ssl01/set6 (MW2+MSSA476) | NEG | NEG | NEG |
|  |  | ssl01/set6 (other alleles) | NEG | NEG | NEG |
|  |  | ssl01/set6 (RF122) | NEG | NEG | NEG |
| ***ssl02*** | staphylococcal superantigen-like protein 2 | ssl02/set7 | AMB | NEG | NEG |
|  |  | ssl02/set7 (MRSA252) | **POS** | **POS** | **POS** |
| ***ssl03*** | staphylococcal superantigen-like protein 3 | ssl03/set8 | NEG | NEG | NEG |
|  |  | ssl03/set8 (MRSA252, SAR0424) | **POS** | **POS** | **POS** |

| **GENE** | **EXPLANATION** | **ALLELE** | **Predicted profile for ATCC 25923 (GenBank CP009361+**  **362)** | **Array hybridization data for ATCC25923-G478** | **Array hybridization data for ATCC25923-G477** |
| --- | --- | --- | --- | --- | --- |
| ***ssl04*** | staphylococcal superantigen-like protein 4 | ssl04/set9 | NEG | NEG | NEG |
|  |  | ssl04/set9 (MRSA252, SAR0425) | **POS** | **POS** | **POS** |
| ***ssl05*** | staphylococcal superantigen-like protein 5 | ssl05/set3 (MRSA252) | **POS** | **POS** | **POS** |
|  |  | ssl05/set3 | NEG | NEG | NEG |
|  |  | ssl05/set3 (RF122) | NEG | NEG | NEG |
| ***ssl06*** | staphylococcal superantigen-like protein 6 | ssl06 (NCTC8325+MW2) | NEG | NEG | NEG |
|  |  | ssl06/set21 | NEG | NEG | NEG |
| ***ssl07*** | staphylococcal superantigen-like protein 7 | ssl07/set1 (MRSA252) | **POS** | **POS** | **POS** |
|  |  | ssl07/set1 (AF188836) | NEG | NEG | NEG |
|  |  | ssl07/set1 | NEG | NEG | NEG |
| ***ssl08*** | staphylococcal superantigen-like protein 8 | ssl08/set12_probe 2 | NEG | NEG | NEG |
|  |  | ssl08/set12_probe 1 | NEG | NEG | NEG |
| ***ssl09*** | staphylococcal superantigen-like protein 9 | ssl09/set5_probe 2 | NEG | NEG | NEG |
|  |  | ssl09/set5_probe 1 | NEG | NEG | NEG |
|  |  | ssl09/set5 (MRSA252) | **POS** | **POS** | **POS** |
| ***ssl10*** | staphylococcal superantigen-like protein 10 | ssl10 (RF122) | NEG | NEG | NEG |
|  |  | ssl10/set4 | NEG | AMB | **POS** |
|  |  | ssl10/set4 (MRSA252) | **POS** | **POS** | **POS** |
| ***hsdS2*** | type I site-specific deoxyribonuclease subunit, 2nd locus | hsdS2-ST5+ST8 | NEG | NEG | NEG |
|  |  | hsdS2-MRSA252 | **POS** | **POS** | **POS** |
|  |  | hsdS2-MW2+476 | NEG | NEG | NEG |
|  |  | hsdS2-RF122 | NEG | NEG | NEG |
| ***ssl11*** | staphylococcal superantigene-like protein 11 | ssl11/set2(MW2/ MSSA476) | NEG | NEG | NEG |
|  |  | ssl11/set2 (MRSA252) | **POS** | **POS** | **POS** |
|  |  | ssl11/set2(Mu50/ N315) | NEG | NEG | NEG |
|  |  | ssl11/set2 (COL) | NEG | NEG | NEG |
| **G7ZRU6** | GNAT acetyltransferase "Argenteus/ST1850-like" | - | NEG | NEG | NEG |
| ***sdrC*** | Ser-Asp rich fibrinogen-/bone sialoprotein binding protein C | sdrC (OtherThan252+RF122) | NEG | NEG | NEG |
|  |  | sdrC (B1) | NEG | NEG | NEG |
|  |  | sdrC (consensus) | **POS** | **POS** | **POS** |
|  |  | sdrC (Mu50) | NEG | NEG | NEG |
|  |  | sdrC (COL) | NEG | NEG | NEG |
|  |  | sdrC (MW2+MRSA252+RF122) | **POS** | AMB | AMB |

| **GENE** | **EXPLANATION** | **ALLELE** | **Predicted profile for ATCC 25923 (GenBank CP009361+**  **362)** | **Array hybridization data for ATCC25923-G478** | **Array hybridization data for ATCC25923-G477** |
| --- | --- | --- | --- | --- | --- |
| ***sdrD*** | Ser-Asp rich fibrinogen-/bone sialoprotein binding protein D | sdrD (consensus) | **POS** | **POS** | **POS** |
|  |  | sdrD (COL+MW2) | NEG | NEG | NEG |
|  |  | sdrD (Mu50) | NEG | NEG | NEG |
|  |  | sdrD (others) | **POS** | **POS** | **POS** |
| ***bbp*** | bone sialoprotein-binding protein | bbp (consensus) | **POS** | **POS** | **POS** |
|  |  | bbp (ST45) | NEG | NEG | NEG |
|  |  | bbp (RF122) | NEG | NEG | NEG |
|  |  | bbp (MRSA252) | **POS** | **POS** | **POS** |
|  |  | bbp (COL+MW2) | NEG | NEG | NEG |
|  |  | bbp (Mu50) | NEG | NEG | NEG |
| ***sarA*** | staphylococcal accessory regulator A | - | **POS** | **POS** | **POS** |
| ***saeS*** | histidine protein kinase, sae locus | - | **POS** | **POS** | **POS** |
| ***gapA*** | glyceraldehyde 3-phosphate dehydrogenase, locus 1 | - | **POS** | **POS** | **POS** |
| ***eno*** | enolase | - | **POS** | **POS** | **POS** |
| ***clfA*** | clumping factor A | clfA (consensus) | **POS** | **POS** | **POS** |
|  |  | clfA (COL+RF122) | AMB | AMB | **POS** |
|  |  | clfA (MRSA252) | **POS** | **POS** | **POS** |
|  |  | clfA (Mu50+MW2) | NEG | NEG | NEG |
| ***vwb*** | van Willebrand factor binding protein | vwb (COL+MW2) | NEG | NEG | NEG |
|  |  | vwb (consensus) | **POS** | **POS** | **POS** |
|  |  | vwb (MRSA252) | **POS** | **POS** | **POS** |
|  |  | vwb (Mu50) | NEG | NEG | NEG |
|  |  | vwb (RF122) | NEG | NEG | NEG |
| ***nuc1*** | thermostable extracellular nuclease | - | **POS** | **POS** | **POS** |
| ***corB*** | putative membrane protein | - | **POS** | **POS** | **POS** |
| ***sspB*** | staphopain B, protease | - | **POS** | **POS** | **POS** |
| ***sspA*** | glutamylendopeptidase | - | **POS** | **POS** | **POS** |
| ***isdA*** | transferrin-binding protein | isdA (consensus) | **POS** | **POS** | **POS** |
|  |  | isdA (MRSA252) | **POS** | **POS** | **POS** |
|  |  | isdA (Other Than MRSA252 ) | NEG | NEG | NEG |
| ***efb*** | fibrinogen binding protein (19 kDa) | efb / fib | NEG | NEG | NEG |
|  |  | efb / fib (MRSA252) | **POS** | **POS** | **POS** |
| ***hla*** | haemolysin alpha | - | **POS** | **POS** | **POS** |
| ***setB3*** | staphylococcal exotoxin-like proteins,  second locus | setB3 | NEG | NEG | NEG |
|  |  | setB3 (MRSA252) | **POS** | **POS** | **POS** |
| ***setB2*** |  | setB2 | NEG | NEG | NEG |
|  |  | setB2 (MRSA252) | **POS** | **POS** | **POS** |
| ***setB1*** |  | setB1 | AMB | **POS** | **POS** |
| ***katA*** | katalase A | - | **POS** | **POS** | **POS** |
| ***mprF*** | defensin resistance protein | mprF (COL+MW2) | NEG | NEG | AMB |
|  |  | mprF (Mu50+MRSA252) | **POS** | **POS** | **POS** |
| ***ebh*** | cell wall associated fibronectin-binding protein | ebh (consensus) | **POS** | **POS** | **POS** |

| **GENE** | **EXPLANATION** | **ALLELE** | **Predicted profile for ATCC 25923 (GenBank CP009361+**  **362)** | **Array hybridization data for ATCC25923-G478** | **Array hybridization data for ATCC25923-G477** |
| --- | --- | --- | --- | --- | --- |
| ***ebpS*** | cell surface elastin binding protein | ebpS (01-1111) | NEG | NEG | NEG |
|  |  | ebpS_probe 612 | **POS** | **POS** | **POS** |
|  |  | ebpS (COL) | NEG | NEG | NEG |
|  |  | ebpS_probe 614 | **POS** | **POS** | **POS** |
| **Q931R4** | major facilitator superfamily transporter | - | **POS** | **POS** | **POS** |
| ***arsB*** | arsenical pump membrane protein | arsB (chromosomal) | **POS** | **POS** | **POS** |
|  |  | arsB (chromosomal-argenteus) | NEG | NEG | NEG |
| ***arsC*** | chromosomal arsenate reductase | - | NEG | **POS** | NEG |
| ***hsdS3*** | type I site-specific deoxyribonuclease subunit, 3rd locus | hsdS3-AllOtherThanRF122+252 | NEG | NEG | NEG |
|  |  | hsdS3-CC51+252 | **POS** | **POS** | **POS** |
|  |  | hsdS3-ST8+ST1+RF122 | NEG | NEG | NEG |
|  |  | hsdS3-MRSA252 | **POS** | **POS** | **POS** |
|  |  | hsdS3-Mu50+N315 | NEG | NEG | NEG |
| ***splE*** | serin protease E | - | **POS** | **POS** | **POS** |
| ***splB*** | serin protease B | - | NEG | NEG | NEG |
| ***splA*** | serin protease A | - | NEG | NEG | NEG |
| ***ear2*** | putative protein, located next to serine protease operon in genomic island beta | - | NEG | NEG | NEG |
| ***lukD*** | leukocidin D component | - | NEG | NEG | NEG |
| ***lukE*** | leukocidin E component | - | NEG | NEG | NEG |
| **Q7A4X2** | putative protein located next to entG and also to lukD/E | - | **POS** | **POS** | **POS** |
| ***seg*** | Enterotoxin G | - | **POS** | **POS** | **POS** |
| ***seln*** | Enterotoxin N | seln (other than RF122) | **POS** | **POS** | **POS** |
|  |  | seln (consensus) | **POS** | **POS** | **POS** |
|  |  | entN-argenteus | NEG | NEG | NEG |
| ***selu*** | Enterotoxin U and/or Y | - | **POS** | **POS** | **POS** |
| ***sei*** | Enterotoxin I | - | **POS** | **POS** | **POS** |
| ***selm*** | enterotoxin M | - | **POS** | **POS** | **POS** |
| ***selo*** | enterotoxin O | - | **POS** | **POS** | **POS** |
| ***vraS*** | sensor protein | - | **POS** | **POS** | **POS** |
| ***sspP*** | staphopain A (staphylopain A), protease | sspP (consensus) | **POS** | **POS** | **POS** |
|  |  | sspP (other than ST93) | **POS** | **POS** | **POS** |
| ***map*** | major histocompatibility complex class II analog protein | map (COL) | NEG | NEG | NEG |
|  |  | map (Mu50+MW2) | NEG | NEG | NEG |
|  |  | map (MRSA252) | **POS** | **POS** | **POS** |
| ***hlb*** | haemolysin beta | hlb | **POS** | **POS** | **POS** |
|  |  | hlb, not truncated | **POS** | **POS** | **POS** |

| **GENE** | **EXPLANATION** | **ALLELE** | **Predicted profile for ATCC 25923 (GenBank CP009361+**  **362)** | **Array hybridization data for ATCC25923-G478** | **Array hybridization data for ATCC25923-G477** |
| --- | --- | --- | --- | --- | --- |
| ***lukX*** | bicomponent leukocidin/ haemolysin toxin family protein (LukA/B, lukG/H) | - | **POS** | **POS** | **POS** |
| ***lukY*** |  | lukY | NEG | NEG | NEG |
|  |  | lukY (ST1850) | NEG | NEG | NEG |
|  |  | lukY (ST30+ST45) | **POS** | **POS** | **POS** |
| ***hld*** | haemolysin delta | - | **POS** | **POS** | **POS** |
| ***agrB*** | accessory gene regulator | agrB-I | NEG | NEG | NEG |
|  |  | agrB-II | NEG | NEG | NEG |
|  |  | agrB-III | **POS** | **POS** | **POS** |
|  |  | agrB-IV | NEG | NEG | NEG |
| ***agrD*** |  | agrD-I | NEG | NEG | NEG |
|  |  | agrD-II | NEG | NEG | NEG |
|  |  | agrD-III | **POS** | **POS** | **POS** |
| ***agrC*** |  | agrC-I | NEG | NEG | NEG |
|  |  | agrC-II | NEG | NEG | NEG |
|  |  | agrC-III | **POS** | **POS** | **POS** |
|  |  | agrC-IV | NEG | NEG | NEG |
| ***czrB*** | zink and cobalt transporter protein | - | **POS** | **POS** | **POS** |
| ***sdrM*** | multidrug efflux protein | sdrM (consensus) | NEG | **POS** | **POS** |
|  |  | sdrM (CC30) | **POS** | **POS** | **POS** |
|  |  | sdrM (argenteus) | NEG | NEG | NEG |
| ***hlIII*** | putative membrane protein | hlIII (consensus) | **POS** | **POS** | **POS** |
|  |  | hlIII (other than RF122) | **POS** | **POS** | **POS** |
| ***hysA2*** | hyaluronate lyase | hysA2 (All others than MRSA252) | NEG | NEG | NEG |
|  |  | hysA2 (MRSA252) | **POS** | **POS** | **POS** |
|  |  | hysA2 (All others than CC8) | **POS** | **POS** | **POS** |
|  |  | hysA2 (COL+USA300+ NCTC825) | NEG | **POS** | **POS** |
| ***sbi*** | IgG-binding protein | - | **POS** | **POS** | **POS** |
| ***hlgA*** | haemolysin gamma, component A | - | **POS** | **POS** | **POS** |
| ***lukS*** | haemolysin gamma / leukocidin, S component | - | **POS** | **POS** | **POS** |
| ***lukF*** | haemolysin gamma / leukocidin, F component | - | **POS** | **POS** | **POS** |
| ***sau*** | type II restriction-modification system endonuclease | sauUSI | **POS** | **POS** | **POS** |
|  |  | sau3AI | NEG | NEG | NEG |
|  |  | sauRF122 | NEG | NEG | NEG |
|  |  | sauSO385 | NEG | NEG | NEG |
| ***sasG*** | Staphylococcus aureus surface protein G | sasG (COL+Mu50) | NEG | NEG | NEG |
|  |  | sasG (MW2) | NEG | NEG | NEG |
|  |  | sasG (Others than MRSA252+RF122) | NEG | NEG | NEG |

| **GENE** | **EXPLANATION** | **ALLELE** | **Predicted profile for ATCC 25923 (GenBank CP009361+**  **362)** | **Array hybridization data for ATCC25923-G478** | **Array hybridization data for ATCC25923-G477** |
| --- | --- | --- | --- | --- | --- |
| ***fnbB*** | fibronectin-binding protein B | fnbB (COL) | NEG | NEG | NEG |
|  |  | fnbB (COL+Mu50+MW2) | AMB | **POS** | AMB |
|  |  | fnbB (Mu50) | AMB | **POS** | **POS** |
|  |  | fnbB (MW2) | NEG | NEG | NEG |
|  |  | fnbB (ST15) | NEG | NEG | NEG |
|  |  | fnbB (ST45-2) | NEG | NEG | NEG |
| ***fnbA*** | fibronectin-binding protein A | fnbA (COL) | NEG | NEG | NEG |
|  |  | fnbA (consensus) | **POS** | **POS** | **POS** |
|  |  | fnbA (MRSA252) | **POS** | **POS** | **POS** |
|  |  | fnbA (Mu50+MW2) | NEG | NEG | NEG |
|  |  | fnbA (RF122) | NEG | NEG | NEG |
| ***crtN*** | dehydrosqualene desaturase | - | **POS** | **POS** | **POS** |
| ***crtM*** | dehydrosqualene synthase from | crtM-nonST93 | **POS** | **POS** | **POS** |
|  | staphyloxanthin biosynthesis operon | crtM-ST93 | NEG | NEG | NEG |
| ***crtP*** | diaponeurosporene oxidase | - | **POS** | **POS** | **POS** |
| ***crtO*** | staphyloxanthin acyltransferase | - | **POS** | **POS** | **POS** |
| ***clfB*** | clumping factor B | clfB (consensus) | **POS** | **POS** | **POS** |
|  |  | clfB (COL+Mu50) | NEG | NEG | NEG |
|  |  | clfB (MW2) | NEG | NEG | NEG |
|  |  | clfB (RF122) | NEG | AMB | **POS** |
| ***aur*** | aureolysin | aur (consensus) | **POS** | **POS** | **POS** |
|  |  | aur (MRSA252) | **POS** | **POS** | **POS** |
|  |  | aur (Other than MRSA252) | NEG | NEG | NEG |
| ***isaB*** | immunodominant antigen B | isaB | NEG | NEG | NEG |
|  |  | isaB (MRSA252) | **POS** | **POS** | **POS** |
| ***icaA*** | intercellular adhesion protein A | - | **POS** | **POS** | **POS** |
| ***icaD*** | biofilm PIA synthesis protein D | - | **POS** | **POS** | **POS** |
| ***icaC*** | intercellular adhesion protein C | - | **POS** | **POS** | **POS** |
| ***cna*** | collagen-binding adhesin | - | **POS** | **POS** | **POS** |

1. **Mobile genetic elements; phage-associated markers**

| **GENE** | **EXPLANATION** | **ALLELE** | **Predicted profile for ATCC 25923 (GenBank CP009361+**  **362)** | **Array hybridization data for ATCC25923-G478** | **Array hybridization data for ATCC25923-G477** |
| --- | --- | --- | --- | --- | --- |
| ***lukF-PV*** | Panton Valentine leukocidin F component | lukF-PV | **POS** | **POS** | **POS** |
| ***lukS-PV*** | Panton Valentine leukocidin S component | lukS-PV | **POS** | **POS** | **POS** |
| ***lukF-PV (P83)*** | F component from hypothetical leukocidin from ruminant strains | lukF-PV (P83) | NEG | NEG | NEG |

| **GENE** | **EXPLANATION** | **ALLELE** | **Predicted profile for ATCC 25923 (GenBank CP009361+**  **362)** | **Array hybridization data for ATCC25923-G478** | **Array hybridization data for ATCC25923-G477** |
| --- | --- | --- | --- | --- | --- |
| ***lukM*** | S component from hypothetical leukocidin from ruminant strains | lukM | NEG | NEG | NEG |
| ***chp*** | chemotaxis-inhibiting protein (CHIPS) | chp | NEG | NEG | NEG |
| ***sak*** | staphylokinase | sak | NEG | NEG | NEG |
| ***scn*** | Staphylococcal complement inhibitor | scn | NEG | NEG | NEG |
| ***sea*** | enterotoxin A | sea | NEG | NEG | NEG |
|  | allele from strain 320E | sea (320E) | NEG | NEG | NEG |
|  | allele from strain N315 =  enterotoxin P | sea (N315) / sep | NEG | NEG | NEG |
| ***see*** | enterotoxin E | see | NEG | NEG | NEG |
| ***sasX / sesI*** | surface-anchored protein X |  | NEG | NEG | NEG |

**3) Mobile genetic elements; other virulence markers**

| **GENE** | **EXPLANATION** | **ALLELE** | **Predicted profile for ATCC 25923 (GenBank CP009361+**  **362)** | **Array hybridization data for ATCC25923-G478** | **Array hybridization data for ATCC25923-G477** |
| --- | --- | --- | --- | --- | --- |
| ***tst1*** | toxic shock syndrome toxin | tst1 (consensus) | NEG | NEG | NEG |
|  |  | tst1 ("human" allele) | NEG | NEG | NEG |
|  |  | tst1 ("bovine" allele, RF122) | NEG | NEG | NEG |
| ***seB*** | enterotoxin B | - | NEG | NEG | NEG |
| ***seC*** | enterotoxin C | - | NEG | NEG | NEG |
| ***seD*** | enterotoxin D | - | NEG | NEG | NEG |
| ***seD2*** | enterotoxin homologue from plasmid EDINA  GenBank: AP003089.1 | - | NEG | NEG | NEG |
| ***seJ*** | enterotoxin J | - | NEG | NEG | NEG |
| ***seK*** | enterotoxin K | - | NEG | NEG | NEG |
| ***seL*** | enterotoxin L | - | NEG | NEG | NEG |
| ***seN2*** | Plasmid-born putative enterotoxin | - | NEG | NEG | NEG |
| ***seQ*** | enterotoxin Q | - | NEG | NEG | NEG |
| ***seR*** | enterotoxin R | - | NEG | NEG | NEG |
| ***seS*** | enterotoxin S | - | NEG | NEG | NEG |
| ***seT*** | enterotoxin T | - | NEG | NEG | NEG |
| ***seU2*** | enterotoxin U2 | - | NEG | NEG | NEG |
| ***seW*** | enterotoxin W from plasmid pWBG762 | - | NEG | NEG | NEG |
| ***edinA*** | epidermal cell differentiation inhibitor | - | NEG | NEG | NEG |
| ***edinB*** | epidermal cell differentiation inhibitor B | - | NEG | NEG | NEG |
| ***edinC*** | epidermal cell differentiation inhibitor C | - | NEG | NEG | NEG |
| ***etA*** | exfoliative toxin serotype A | - | NEG | NEG | NEG |
| ***etB*** | exfoliative toxin serotype B | - | NEG | NEG | NEG |
| ***etD*** | exfoliative toxin D | - | NEG | NEG | NEG |
| ***etD2*** | exfoliative toxin homologue from CC130 and CC152 | - | NEG | NEG | NEG |
| ***bap*** | surface protein involved in biofilm formation | - | NEG | NEG | NEG |
| ***sagD*** | putative bacteriocin biosynthesis associated protein | - | NEG | NEG | NEG |

Note: *egc* enterotoxin genes are listed under “1) Core genomic markers” as well as *sea* and *see* under “2) Mobile genetic elements; phage-associated markers”

**4) Mobile genetic elements; SCC-associated markers**

| **GENE** | **EXPLANATION** | **ALLELE** | **Predicted profile for ATCC 25923 (GenBank CP009361+**  **362)** | **Array hybridization data for ATCC25923-G478** | **Array hybridization data for ATCC25923-G477** |
| --- | --- | --- | --- | --- | --- |
| **adhC** | Alcohol dehydrogenase, zinc-containing | adhC (FPR3757) | NEG | NEG | NEG |
| ***arcA-*SCC** | Arginine deiminase | - | NEG | NEG | NEG |
| ***arcB-*SCC** | Ornithine carbamoyltransferase | - | NEG | NEG | NEG |
| ***arcC-*SCC** | Carbamate kinase | - | NEG | NEG | NEG |
| ***arcD-*SCC** | Arginine/ornithine antiporter | - | NEG | NEG | NEG |
| ***arsB-*SCC** | Arsenical pump membrane protein | - | NEG | NEG | NEG |
| ***arsC-*SCC** | Arsenate reductase | - | NEG | NEG | NEG |
| **B2Y834** | Abortive phage resistance protein | - | NEG | NEG | NEG |
| **B6VQU0** | Putative protein | - | NEG | NEG | NEG |
| ***blaZ* (SCC*mec* XI)** | Beta-lactamase from SCCmec XI | - | NEG | NEG | NEG |
| **C5QAP8** | Putative protein | C5QAP8 (SCCmec XI) | NEG | NEG | NEG |
| ***cadD*** | Cadmium transport protein D | *cadD* (R35) | NEG | NEG | NEG |
| ***cadX*** | Putative regulator of cadmium efflux | cadX (JCSC6943) | NEG | NEG | NEG |
| ***cap 1*** | Locus encoding SCC associated capsule type 1 |  | NEG | NEG | NEG |
| ***cas1*** | CRISPR-associated endonuclease 1 | cas1 (M06-0171) | NEG | NEG | NEG |
|  |  | cas1 (MSHR1132) | NEG | NEG | NEG |
| ***ccrA-1*** | Cassette chromosome recombinase A, type 1 | - | NEG | NEG | NEG |
| ***ccrA-2*** | Cassette chromosome recombinase A, type 2 | - | NEG | NEG | NEG |
| ***ccrA-3*** | Cassette chromosome recombinase A, type 3 | - | NEG | NEG | NEG |
| ***ccrA-4*** | Cassette chromosome recombinase A, type 4 | - | NEG | NEG | NEG |
| ***ccrAA*** | “Cassette chromosome recombinase AA” | - | NEG | NEG | NEG |
| ***ccrB-1*** | Cassette chromosome recombinase B, type 1 | - | NEG | NEG | NEG |
| ***ccrB-2*** | Cassette chromosome recombinase B, type 2 | - | NEG | NEG | NEG |
| ***ccrB-3*** | Cassette chromosome recombinase B, type 3 | - | NEG | NEG | NEG |
| ***ccrB-4*** | Cassette chromosome recombinase B, type 4 | - | NEG | NEG | NEG |
| ***ccrC*** | Cassette chromosome recombinase C | - | NEG | NEG | NEG |

| **GENE** | **EXPLANATION** | **ALLELE** | **Predicted profile for ATCC 25923 (GenBank CP009361+**  **362)** | **Array hybridization data for ATCC25923-G478** | **Array hybridization data for ATCC25923-G477** |
| --- | --- | --- | --- | --- | --- |
| ***copA2-*SCC** | Copper exporting ATPase | - | NEG | NEG | NEG |
| ***cstB-SCC1*** | CsoR-like sulfur transferase-regulated genes B/metallo-beta-lactamase superfamily protein. Pseudogene containing two stop codons | - | NEG | NEG | NEG |
| ***cstB-SCC2*** | CsoR-like sulfur transferase-regulated genes B/metallo-beta-lactamase superfamily protein. | - | NEG | NEG | NEG |
| ***czrC*** | Cadmium and zinc resistance gene C, heavy metal translocating P-type ATPase | - | NEG | NEG | NEG |
| ***D1GU38*** | Putative protein | - | NEG | NEG | NEG |
| ***D1GU55*** | Putative membrane protein | - | NEG | NEG | NEG |
| ***D3JD07*** | Putative protein | - | NEG | NEG | NEG |
| ***Delta mecR1*** | Truncated methicillin resistance operon repressor | - | NEG | NEG | NEG |
| ***DUF1958*** | Putative protein | - | NEG | NEG | NEG |
| ***fusC*** | SCC-associated fusidic acid resistance gene | - | NEG | NEG | NEG |
| ***kdpA-SCC*** | Potassium-translocating ATPase A, chain 2 | - | NEG | NEG | NEG |
| ***kdpB-SCC*** | Potassium-transporting ATPase B, chain 1 | - | NEG | NEG | NEG |
| ***kdpC-SCC*** | Potassium-translocating ATPase C, chain 2 | - | NEG | NEG | NEG |
| ***kdpD-SCC*** | Sensor kinase protein | - | NEG | NEG | NEG |
| ***kdpE-SCC*** | KDP operon transcriptional regulatory protein | - | NEG | NEG | NEG |
| ***mco-SCC*** | Multi copper oxidase | - | NEG | NEG | NEG |
| ***mecA*** | Modified penicillin binding protein (PBP2a) | - | NEG | NEG | NEG |
| ***mecC*** | Alternate gene encoding a modified penicillin binding protein | - | NEG | NEG | NEG |
| ***mecI*** | Methicillin-resistance regulatory protein | - | NEG | NEG | NEG |
| ***mecR1*** | Methicillin resistance operon repressor 1 | - | NEG | NEG | NEG |
| ***merA*** | Mercury reductase | - | NEG | NEG | NEG |
| ***merB*** | Alkylmercury lyase | - | NEG | NEG | NEG |
| ***mvaS-SCC*** | Truncated 3-hydroxy-3-methylglutaryl CoA synthase | - | NEG | NEG | NEG |
| ***opp3B*** | Oligopeptide permease, channel-forming protein | opp3B (C427) | NEG | NEG | NEG |
|  |  | opp3B (FPR3757) | NEG | NEG | NEG |
| ***opp3C*** | Oligopeptide permease, channel-forming protein | opp3C (C427) | NEG | NEG | NEG |
|  |  | opp3C (FPR3757) | NEG | NEG | NEG |
| ***pls-SCC*** | Plasmin-sensitive surface protein | pls-SCC (COL) | NEG | NEG | NEG |
| ***PSM-mec*** | Phenol soluble modulin from SCCmec | - | NEG | NEG | NEG |
| ***Q3YK51*** | Putative protein | - | NEG | NEG | NEG |
| ***Q4LAG7*** | Putative protein located within SCCmec V/SCCfus | - | NEG | NEG | NEG |
| ***Q8CU82*** | Putative protein | - | NEG | NEG | NEG |
| ***Q933A2*** | Putative ADP-ribosyltransferase | - | NEG | NEG | NEG |

| **GENE** | **EXPLANATION** | **ALLELE** | **Predicted profile for ATCC 25923 (GenBank CP009361+**  **362)** | **Array hybridization data for ATCC25923-G478** | **Array hybridization data for ATCC25923-G477** |
| --- | --- | --- | --- | --- | --- |
| ***Q93IB7*** | LytTR domain DNA-binding regulator | - | NEG | NEG | NEG |
| ***Q9S0M4*** | Putative protein | - | NEG | NEG | NEG |
| ***Q9XB68-dcs*** | Located at the end of SCCmec next to orfX. | - | NEG | NEG | NEG |
| ***SCC terminus*** | SCC integration site alternate to dcs | SCC terminus 01 | NEG | NEG | NEG |
|  |  | SCC terminus 02 | NEG | NEG | NEG |
|  |  | SCC terminus 03 | NEG | NEG | NEG |
|  |  | SCC terminus 04 | NEG | NEG | NEG |
|  |  | SCC terminus 05 | NEG | NEG | NEG |
|  |  | SCC terminus 06 | NEG | NEG | NEG |
|  |  | SCC terminus 07 | NEG | NEG | NEG |
|  |  | SCC terminus 09 | NEG | NEG | NEG |
|  |  | SCC terminus 10 | NEG | NEG | NEG |
|  |  | SCC terminus 11 | NEG | NEG | NEG |
|  |  | SCC terminus 12 | NEG | NEG | NEG |
|  |  | SCC terminus 13 | NEG | NEG | NEG |
|  |  | SCC terminus 14 | NEG | NEG | NEG |
| ***speG*** | Spermidine N-acetyltransferase | speG (FPR3757) | NEG | NEG | NEG |
| ***tirS*** | Staphylococcal TIR-protein binding protein | - | NEG | NEG | NEG |
| ***ugpQ*** | Glycerophosphoryl diester phosphodiesterase | - | NEG | NEG | NEG |
| ***xylR/ mecR2*** | Methicillin resistance operon repressor 2, Homolog of xylose repressor | - | NEG | NEG | NEG |
| ***ydhK*** | Putative lipoprotein | ydhK (FPR3757) | NEG | NEG | NEG |
| ***yeeA*** | Putative DNA methyltransferase | - | NEG | NEG | NEG |

**5) Mobile genetic elements; other resistance markers**

| **GENE** | **EXPLANATION** | **ALLELE** | **Predicted profile for ATCC 25923 (GenBank CP009361+**  **362)** | **Array hybridization data for ATCC25923-G478** | **Array hybridization data for ATCC25923-G477** |
| --- | --- | --- | --- | --- | --- |
| ***aacA-aphD*** | bifunctional enzyme Aac/Aph, gentamicin resistance | - | NEG | NEG | NEG |
| ***aad6*** | streptomycin adenyltransferase | - | NEG | NEG | NEG |
| ***aadA*** | streptomycin/spectinomycin 3' adenyltransferase | - | NEG | NEG | NEG |
| ***aadD*** | aminoglycoside adenyltransferase,tobramycin resistance | - | NEG | NEG | NEG |
| ***aadE*** | streptomycin aminoglycoside 6-adenyltransferase | aadE | NEG | NEG | NEG |
|  |  | aadE-C2944 | NEG | NEG | NEG |
| ***ant9*** | Streptomycin 3''-adenylyltransferase | ant9 | NEG | NEG | NEG |
|  |  | ant9-C2944 | NEG | NEG | NEG |

| **GENE** | **EXPLANATION** | **ALLELE** | **Predicted profile for ATCC 25923 (GenBank CP009361+**  **362)** | **Array hybridization data for ATCC25923-G478** | **Array hybridization data for ATCC25923-G477** |
| --- | --- | --- | --- | --- | --- |
| ***aphA3*** | 3'5'-aminoglycoside phosphotransferase, neo-/ kanamycin resistance | - | NEG | NEG | NEG |
| ***apmA*** | aminocyclitol acetyltransferase, confers apramycin resistance | - | NEG | NEG | NEG |
| ***arsB (plasmid)*** | arsenical pump membrane protein | arsB (plasmidic) | **POS** | **POS** | **POS** |
| ***blaI*** | beta lactamase repressor (inhibitor) | - | NEG | NEG | NEG |
| ***blaR*** | beta-lactamase regulatory protein | - | NEG | NEG | NEG |
| ***blaZ*** | beta-lactamase | - | NEG | NEG | NEG |
| ***ble*** | Bleomycin resistance | - | NEG | NEG | NEG |
| ***cadA*** | cadmium efflux adenosine triphosphatase | cadA (pTW20) | NEG | NEG | NEG |
|  |  | cadA (Sepi) | NEG | NEG | NEG |
|  |  | cadA (TN554) | **POS** | **POS** | **POS** |
|  |  | cadC (pI258) | NEG | NEG | NEG |
|  |  | cadC (TN554) | **POS** | **POS** | **POS** |
| ***cadD*** | cadmium transport protein D |  | NEG | NEG | NEG |
| ***cadX (plasmid)*** | putative regulator of cadmium efflux | cadX (plasmidic) | NEG | NEG | NEG |
| ***cat*** | chloramphenicol acetyltransferase | cat (pC221) | NEG | NEG | NEG |
|  |  | cat (pc223) | NEG | NEG | NEG |
|  |  | cat (pMC524) | NEG | NEG | NEG |
|  |  | cat (pSBK203R) | NEG | NEG | NEG |
|  |  | cat (Saga) | NEG | neg | NEG |
| ***cfr*** | 23S rRNA methyltransferase | cfr | NEG | NEG | NEG |
| ***copA2 (plasmid)*** | Plasmid-born copper exporting ATPase | copA2_pla | **POS** | **POS** | **POS** |
| ***dfrA*** | dihydrofolate reductase type 1 | dfrA | NEG | NEG | NEG |
| ***dfrG*** | dihydrofolate reductase | dfrG | NEG | NEG | NEG |
|  |  | dfrG-D | NEG | NEG | NEG |
|  |  | dfrG-K | NEG | NEG | NEG |
|  |  | dfrG-Tn559 | NEG | NEG | NEG |
| ***ermA*** | rRNA adenine N-6-methyl-transferases conferring erythromycin/clindamycin resistance | ermA | NEG | NEG | NEG |
|  |  | ermA-43 | NEG | NEG | NEG |
| ***ermB*** |  | ermB | NEG | NEG | NEG |
| ***ermC*** |  | ermC | NEG | NEG | NEG |
|  |  | ermC-GM | NEG | NEG | NEG |
| ***ermF*** |  | ermF | NEG | NEG | NEG |
| ***ermT*** |  | ermT | NEG | NEG | NEG |
| ***fexA*** | chloramphenicol/florfenicol exporter | fexA | NEG | NEG | NEG |
| ***fosB*** | metallothiol transferase | fosB | **POS** | **POS** | **POS** |
| ***fosB (plasmid)*** | metallothiol transferase | fosB (plasmidic) | NEG | NEG | NEG |
| ***fusB/far1*** | fusidic acid resistence | far1 | NEG | NEG | NEG |

| **GENE** | **EXPLANATION** | **ALLELE** | **Predicted profile for ATCC 25923 (GenBank CP009361+**  **362)** | **Array hybridization data for ATCC25923-G478** | **Array hybridization data for ATCC25923-G477** |
| --- | --- | --- | --- | --- | --- |
| ***linA/lnuA*** | Lincosamid-Nucleotidyltransferase | linA/lnuA | NEG | NEG | NEG |
| ***lsa-B*** | lincosamide ABC transporter | lsa-B | NEG | NEG | NEG |
| ***lsa-E*** | lincosamide ABC transporter | lsa-E | NEG | NEG | NEG |
| ***mco*** | multi copper oxidase | mco -plasmid (MRSA252) | **POS** | **POS** | **POS** |
| ***mefA*** | macrolide efflux protein A | mefA | NEG | NEG | NEG |
| ***mph(C)*** | probable lysylphos-phatidyl-glycerol synthetase | mph(C) | NEG | NEG | NEG |
| ***msrA*** | energy-dependent efflux of erythro-mycin | msrA | NEG | NEG | NEG |
| ***mupA*** | mupirocin resistence protein | mupA | NEG | NEG | NEG |
| ***mupB*** | plasmidic isoleucyl-tRNA synthase | mupB | NEG | NEG | NEG |
| **Q2YUB3** | multidrug resistance transporter | Q2YUB3 | NEG | NEG | NEG |
|  |  | Q2YUB3 (Sepi) | NEG | NEG | NEG |
|  |  | Q2YUB3 (RF122) | NEG | NEG | NEG |
|  |  | Q2YUB3 (Swar) | NEG | NEG | NEG |
| ***qacA*** | quaternary ammonium compound resistance protein A | qacA | NEG | NEG | NEG |
| ***qacC*** | quaternary ammonium compound resistance protein C | qacC | NEG | NEG | NEG |
|  |  | qacC (equine) | NEG | NEG | NEG |
|  |  | qacC (SA5) | NEG | NEG | NEG |
|  |  | qacC (Ssap) | NEG | NEG | NEG |
|  |  | qacC (ST94) | NEG | NEG | NEG |
| ***sat*** | streptothricine-acetyltransferase | sat | NEG | NEG | NEG |
| ***smr*** | quaternary ammonium resistance protein | smr (AL1) | NEG | NEG | NEG |
|  |  | smr (KACC16562) | NEG | NEG | NEG |
|  |  | smr (OJ82) | NEG | NEG | NEG |
|  |  | smr (VCU012) | NEG | NEG | NEG |
| ***tetK*** | Tetracycline resistance gene | tetK | NEG | NEG | NEG |
| ***tetL*** | Tetracycline resistance gene | tetL | NEG | NEG | NEG |
| ***tetM*** | Tetracycline resistance gene | tetM | NEG | NEG | NEG |
|  |  | tetM-O | NEG | NEG | NEG |
|  |  | tetM-S | NEG | NEG | NEG |
| ***vanA*** | vancomycin resistance gene | vanA | NEG | NEG | NEG |
| ***vanB*** | vancomycin resistance gene from enterococci and Clostridium | vanB | NEG | NEG | NEG |
| ***vanZ*** | teicoplanin resistance gene from enterococci | vanZ | NEG | NEG | NEG |
| ***vat(A)*** | virginiamycin A acetyltransferase | vat(A) | NEG | NEG | NEG |
| ***vat(B)*** | acetyltransferase inactivating streptogramin A | vat(B) | NEG | NEG | NEG |
| ***vga(A)*** | ATP binding protein, streptogramin-A-resistance | vga(A) | NEG | NEG | NEG |
|  |  | vga(A) (BM 3327) | NEG | NEG | NEG |
| ***vgaB*** | streptogramin A ABC transporter | vgaB | NEG | NEG | NEG |
| ***vgaC*** | streptogramin A ABC transporter | vgaC | NEG | NEG | NEG |
| ***vgaD*** | streptogramin A ABC transporter | vgaD | NEG | NEG | NEG |
| ***vgaE*** | streptogramin A ABC transporter | vgaE | NEG | NEG | NEG |
| ***vgb*** | virginiamycin B hydrolase | vgb | NEG | NEG | NEG |
