# Supplementary material for: PVL overexpression due to genomic rearrangements and mutations in the S. aureus reference strain ATCC25923
Source: BMC Res Notes. 2017 Nov 7;10:576. doi: 10.1186/s13104-017-2891-3 (PMC5678758; doi:10.1186/s13104-017-2891-3)
Supplement: Supplementary file 2 — Additional file 2. Primers used for filling the gaps between the NGS contigs by sequencing of PCR products. [file 13104_2017_2891_MOESM2_ESM.docx]

**Additional file 2: Primers used for filling the gaps between the NGS contigs by sequencing of PCR products.**

| **Designation** | **Sequence (5´-3´)** | **Position in genome** | **Polymerase used** | **Sequencing**  **method** |
| --- | --- | --- | --- | --- |
| G477-Gap1-F | CCTGTCCAGAAAAATAATAAG | 996397..996417 | GoTaq MDx Hot Start Polymerase, Promega; cat. nr. D6005 | Sanger |
| G477-Gap1-R | CGCTATAGCTAATAATACAAC | 996885..996905 |  |  |
| G477-Gap2-F | AATAACTATCCATCTTTAACAAG | 2037513..2037535 | GoTaq MDx Hot Start Polymerase | Sanger |
| G477-Gap2-R | AATATGAAAGAGTAGTAACTTC | 2037882..2037903 |  |  |
| G477-Gap3-F | TAATCGTAGTTTCGTTGAAATG | 2414727..2414748 | GoTaq MDx Hot Start Polymerase | Sanger |
| G477-Gap3-R | AGTCATCATCATGATTACTAG | 2415275..2415295 |  |  |
| G478-Gap1-F | TTCCATTCTTTGAGCGACTA | 48636..48655 | GoTaq MDx Hot Start Polymerase | Sanger |
| G478-Gap1-R | ACAAATAAAACGAAATTCATGG | 49092..49113 |  |  |
| G478-Gap2-F | TAATCAATATGTCAATTTAGAATA | 1122462..1122485 | GoTaq MDx Hot Start Polymerase | Sanger |
| G478-Gap2-R | TAGAAATGCGATATAAAATAGTT | 1123091..1123113 |  |  |
| G478-Gap3-F | GATAGAGATTTCTAAAATTGCT | 1301879..1301900 | GoTaq MDx Hot Start Polymerase | Sanger |
| G478-Gap3-R | TTAAAAGTAAACTTTCTTTATCC | 1302437..1302459 |  |  |
| G478-Gap4-F | AAAAATGATAAGAATGGTTAAAG | 1292564..1292586 | GoTaq MDx Hot Start Polymerase | Sanger |
| G478-Gap4-R | ATACATTGTTTCTTTGTAGTTG | 1293265..1293286 |  |  |
| G478-Gap5-F | TACAAGTGTAAATAAACTTGCT | 1845722..1845743 | GoTaq MDx Hot Start Polymerase | Sanger |
| G478-Gap5-R | CTTTATTGTTGAAGATGACGA | 1846206..1846226 |  |  |
| G478-Gap6-F | GTTGCTTAAATTCTTCCACG | 2102831..2102850 | GoTaq Long PCR Master Mix, Promega; cat. nr. M4021 | NGS (MiSeq) |
| G478-Gap6-R | AAAATTAGGCGTCTATTATATG | 2116539..2116560 |  |  |
